# [Expression of Concern] Rap2B promotes the proliferation and migration of human glioma cells via activation of the ERK pathway

**DOI:** 10.3892/ol.2026.15645

**Published:** 2026-05-11

**Authors:** Guohong Shi, Zhen Zhang

Oncol Lett 21: 314, 2021; DOI: 10.3892/ol.2021.12575

Following the publication of the above paper, it has been drawn to the Editor's attention by a concerned reader that, regarding the Transwell invasion assay data shown in Fig. 3A on p. 6, the Control and si-Rap2B data panels contained an overlapping section, suggesting that these data panels, which were intended to show the results of differently performed experiments, had been derived from the same original source. A subsequent analysis of the data in this paper undertaken by the Editorial Office revealed that the Control data panels comparing Fig. 3A with 3B also contained an overlapping section.

The authors have been contacted by the Editorial Office to offer an explanation for these apparent anomalies in the presentation of the data in this paper, and we are awaiting their response. Owing to the fact that the Editorial Office has been made aware of potential issues surrounding the scientific integrity of this paper, we are issuing an Expression of Concern to notify readers of this potential problem while the Editorial Office continues to investigate this matter further.

